# In Vitro Neuroprotective and Anti-Inflammatory Activities of Natural and Semi-Synthetic Spirosteroid Analogues

**DOI:** 10.3390/molecules21080992

**Published:** 2016-07-29

**Authors:** Laura García-Pupo, Armando Zaldo-Castro, Vassiliki Exarchou, Juan Enrique Tacoronte-Morales, Luc Pieters, Wim Vanden Berghe, Yanier Nuñez-Figueredo, René Delgado-Hernández

**Affiliations:** 1 Centro de Investigación y Desarrollo de Medicamentos (CIDEM), Ave 26, No. 1605 Boyeros y Puentes Grandes, CP 10600 La Habana, Cuba; laura.garcia@cidem.cu (L.G.-P.); yanier.nunez@cidem.cu (Y.N.F.); 2 Centro de Estudios de Productos Naturales, Facultad de Química, Universidad de la Habana, Zapata s/n entre G y Carlitos Aguirre, Vedado, Plaza de la Revolución, CP 10400 La Habana, Cuba; zaldo@fq.uh.cu (A.Z.-C.); jetacoronte@yahoo.com (J.E.T.-M.); 3 Natural Products & Food Research & Analysis (NatuRA), Department of Pharmaceutical Sciences, University of Antwerp, Universiteitsplein 1, PC 2610 Antwerp, Belgium; vasiliki.exarchou@uantwerpen.be (V.E.); luc.pieters@uantwerpen.be (L.P.); 4 Protein Chemistry, Proteomics and Epigenetic Signaling (PPES), Department of Biomedical Sciences, University of Antwerp, Universiteitsplein 1, PC 2610 Antwerp, Belgium; wim.vandenberghe@uantwerpen.be; 5 Centro de Estudios Investigaciones y Evaluaciones Biológicas (CEIEB), Instituto de Farmacia y Alimentos (IFAL), Universidad de la Habana, Calle 222 #2317/23 y 31 La Coronela, La Lisa, CP 13600 La Habana, Cuba

**Keywords:** spirosteroids, excitotoxicity, neuroprotection, inflammatory mediators

## Abstract

Two spirosteroid analogues were synthesized and evaluated for their in vitro neuroprotective activities in PC12 cells, against glutamate-induced excitotoxicity and mitochondrial damage in glucose deprivation conditions, as well as their anti-inflammatory potential in LPS/IFNγ-stimulated microglia primary cultures. We also evaluated the in vitro anti-excitotoxic and anti-inflammatory activities of natural and endogenous steroids. Our results show that the plant-derived steroid solasodine decreased PC12 glutamate-induced excitotoxicity, but not the cell death induced by mitochondrial damage and glucose deprivation. Among the two synthetic spirosteroid analogues, only the (25*R*)-5α-spirostan-3,6-one (**S15**) protected PC12 against ischemia-related in vitro models and inhibited NO production, as well as the release of IL-1β by stimulated primary microglia. These findings provide further insights into the role of specific modifications of the A and B rings of sapogenins for their neuroprotective potential.

## 1. Introduction

Cerebrovascular diseases, like cerebral ischemia, represent one of the first causes of mortality and morbidity worldwide, which generate high cost by means of medical and social care [1]. Focal cerebral ischemia occurs after the permanent or transient occlusion of a brain artery or its communicants, due to blood thrombus or embolism, which immediately causes a decrease in blood flow and therefore in oxygen and glucose to a defined brain region [2,3]. Consequently, brain tissue and neurons start to die because of glutamate-induced excitotoxicity signaling, ionic and oxidative imbalance, mitochondrial damage and inflammatory response [4,5]. Several in vitro models have been employed for mimicking the ischemia conditions, especially glutamate-induced excitotoxicity, and to evaluate therapeutic candidates [6,7]. In PC12 cells, exposure to high doses of glutamate decrease their survival [8,9], and this toxic effect may occur via two different process. The classical pathway, known as excitotoxicity, occurs through the activation of NMDA and non-NMDA glutamatergic receptors [10] and subsequent calcium influx into the cell [11]. The oxidative glutamate toxicity pathway is a transporter-mediated type of cell death [12], which requires the cellular expression of the cystine/glutamate antiporter system [8]. This pathway has been described in primary neuronal cultures, neuronal cell lines [13] and in brain tissue slices [14]. It has been extensively demonstrated that mitochondria are involved in the control of neuronal Ca^2+^ homeostasis [15], neuronal Ca^2+^ signaling [16] and Ca^2+^-dependent exocytosis [17]. Mitochondrial Ca^2+^ overload and dysfunction, due to excessive Ca^2+^ entry through over-activated glutamate receptors, is a crucial early event in the excitotoxic cascade that follows ischemic ictus [18]. In isolated mitochondria, Ca^2+^ overload can evoke sustained mtPTP (mitochondrial transient permeability transition pore) opening and cytochrome c release. Opening of the mtPTP causes a massive swelling of mitochondria coupled with the collapse of the mitochondrial membrane potential [18]. Despite the detailed description of mechanisms and mediators related to the pathophysiology of cerebral ischemia and stroke, to date, almost all neuroprotective candidates have failed in clinical trials [2,19].

Sex hormones are essential regulators of survival, differentiation, neurotransmitter synthesis and the synaptic plasticity of neurons and glia in brain and spinal cord [20]. In the 1990s, several studies demonstrated the neuroprotective effects of estrogens in animal models of cerebral ischemia [21]. In parallel, some estrogenic steroids, such as 17β-estradiol (E2), showed neuroprotective properties related to excitotoxicity antagonism by free radical scavenging and antioxidant activity [22,23]. The mechanisms involved in the neuroprotective effects of estrogen include intrinsic antioxidant activities, as well as estrogen receptor (ER)-dependent genomic and fast non-genomic signaling pathways. Almost two decades ago, the importance of E2 antioxidant activity for its neuroprotective potential was demonstrated in cell-based in vitro models of glutamate-induced excitotoxicity and associated with specific structural features [22,24,25]. This antioxidant potential relies on the phenolic ring present in the structure of the estradiol molecule, which also affords the redox cycling activity in neurons [26]. Concerning the ER-dependent pathways, it is currently being accepted that several estrogen receptors are involved in the neuroprotective effects of estradiol and not only ERα, as was initially thought (reviewed in [27,28]). It has been previously demonstrated that E2 prevents ischemia-induced downregulation of the pro-survival bcl-2 gene, while it attenuates c-fos upregulation, promoting cell survival and decreasing neuronal programmed cell death. Furthermore, E2 enhances the activation of Akt kinase, which is an important mediator of cell survival signaling pathways [27]. On the other hand estrogens suppress neuroinflammation, which is a hallmark of many neurodegenerative diseases, such as stroke, Parkinson’s and Alzheimer’s diseases. Physiological levels of E2 suppress both microglial activation and iNOS upregulation, as well as several proinflammatory cytokines, via ERα and suppression of the NFκB transcription factor [27,29]. Some other studies with inhibitors for kinases involved in classical ER and membrane-coupled ER signaling in primary neuronal cultures demonstrated that the activity of estrogens and some neuroprotective benzothiophene selective estrogen receptor modulators is mediated via ER-independent and G-protein-coupled responses through the PI3K, Src and ERK-dependent signaling pathways [30].

Furthermore, sulfated neurosteroids, like dehydroepiandrosterone sulfate and pregnenolone sulfate, have been defined as allosteric regulators of ionotropic receptors activity [31,32]. However clinical trials with post-menopausal women also showed an increase of cardiovascular risk, as adverse effects in the use of estrogenic compounds [33,34]. Therefore, the design, characterization and isolation or synthesis of molecular entities with neuroprotective activity is still an interesting topic of drug research and development. Spiro compounds are widely distributed in nature as polyether antibiotics, talaromycines, pheromones, milbemycines, avermectines and also steroidal spirostanes. All of these compounds, having a spiroacetal moiety, show a high and diverse biological activity [35]. In addition, the neuroprotective potential of naturally-occurring sapogenins, such as solasodine, as neuroprotective and neurogenic compounds has been described [36,37]. Owing to their peculiar structural features, we synthesized various spirosteroids with diosgenin as the starting product, and their synthetic sequence can be summarized in the reactions of the oxidation/dehydration of the C3, C5 or C6 positions in the A and B rings.

The aim of the present investigation was an in vitro evaluation of the potential neuroprotective and/or anti-inflammatory effects of a series of natural spirosteroids and their semi-synthetic analogues, providing new insights into their potential as neuroactive molecules [6,7,8]. We followed a modified Aburatani method and used Jones reagent to obtain two spirosteroids analogs, with different functional groups at the C3 position, together with an oxygenated substituent at positions C5 and C6, in order to test the hypothesis that modifications in those positions influences the neuroprotective potential of the spirosteroid derivatives. We also aimed to study the in vivo estrogenic activity of the most active spirosteroid analogue in the in vitro models, as a criterion for low potential hormonal side effects.

## 2. Results and Discussion

### 2.1. Chemistry

**Scheme 1:**
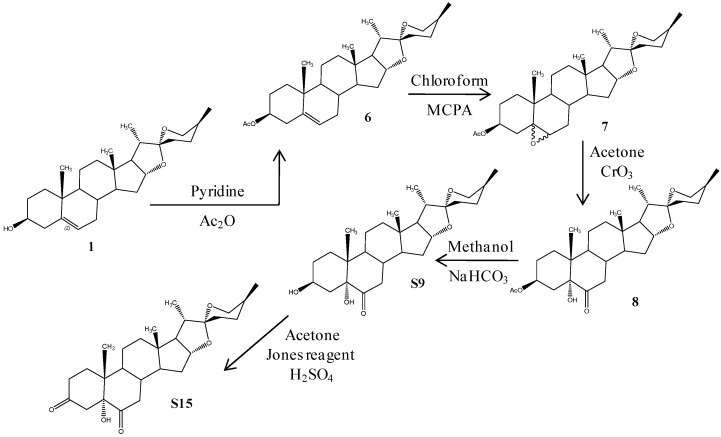
Synthetic route of sapogenin derivatives. MCPA, meta-chloro-perbenzoic acid. **1** Diosgenin, **6** (25*R*)-5-Spirost-3β-ol-3-acetate, **7** (25*R*)-5,6-Epoxy-spirost-3β-ol-3-acetate, **8** (25*R*)-Spirost-3β,5α-dihydroxy-6-one-3-acetate, **S9** (25*R*)-Spirost-3β,5α-dihydroxy-6-one, **S15** (25*R*)-Spirost-5α-hydroxy-3,6-dione.

Several spirosteroid derivatives were prepared according to the synthetic pathway depicted in Scheme 1. The common raw material for all of these compounds was diosgenin, a plant-derived and commercially available compound (Phytomarker Ltd., Tianjin, China) with a purity of more than 95% (Figure S1).

Considering previous evidence about the critical role of oxygenated functional groups at position C3 in the A ring of steroids [38], we then move onto synthesized 3-oxo derivatives of diosgenin. Treatment of diosgenin **1** with acetic anhydride in pyridine produced diosgenin acetate **6** with a 95% yield. Subsequently, the reaction of diosgenin acetate **6** with meta-chloroperbenzoic acid at room temperature, after several washes with sodium sulfate and carbonate salts, generated a mixture of the α/β5,6-epoxy Compound **7** with an 85% total yield. Aqueous CrO_3_ oxidative opening of this mixture, without previous separation and dissolved in warm acetone, produced Compound **8**, after re-crystallization from methanol, with an 80% yield, which is similar to previous results in the literature [39]. Hydrolysis of **8** under basic conditions with refluxing NaHCO_3_ yielded the diol **S9** with a 95% total yield. After normalization of the peak area detected by HPLC-DAD, the purity of Compound **S9** was determined to be more than 97% (Figure S2). On the other hand, 5-ol-diketone **S15** was synthesized by oxidation of the secondary alcohol in position C3 of Compound **S9**, with Jones reagent in the presence of sulfuric acid, which also caused the dehydration of **S9** at the most reactive tertiary alcohol in the C5 position. Therefore, we also obtained the minor product 4-en-3,6-dione, with the double bond stabilized by the resonance with the carbonyl groups at the C3 and C6 positions. Purification by column chromatography gave pure Compound **S15** with an 81.5% yield and 90% purity according to HPLC-DAD (Figure S3).

The structures of Compounds **S9** and **S15** were identified by spectroscopic analysis, including ^1^H-NMR spectra and distortionless enhancement by polarization transfer (DEPT)^13^C-NMR spectra. In order to identify previous published information, concerning the chemical structures of the obtained compounds, an extensive search was done in the SciFinder database [40]. The previously-reported carbon NMR data for Compounds **S9** [41] and **S15** [42] are in concordance with the carbon spectroscopic data we obtained (Figures S4 and S5).

### 2.2. Biological Activity

#### 2.2.1. Treatments to PC12 Cell Cultures

**Figure 1:**
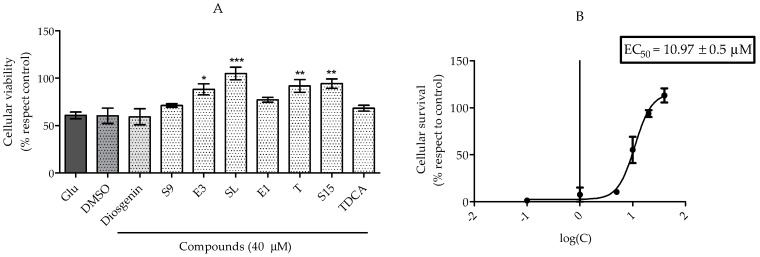
Anti-excitotoxic activity of compounds in glutamate-induced cell death. (**A**) PC12 cell cultures were exposed to an established excitotoxic concentration of glutamate (Glu) (20 mM), and some were also treated with each compound (**S9** and **S15** semi-synthetic derivatives; E3, estriol; SL, solasodine; E1, estrone; T, testosterone; TDCA, taurodeoxycholic acid) or solvent control (DMSO). After 24 h of exposition to the damage and treatment, cellular viability was determined by the MTT assay; (**B**) Dose-response of Compound **S15** in the same model of glutamate-induced excitotoxicity. Data are represented as the percent with respect to the untreated cells, representing the highest cell viability. * *p* < 0.05, ** *p* < 0.01 and *** *p* < 0.001, according to one-way ANOVA and Tukey’s *post-hoc* test.

**Figure 2:**
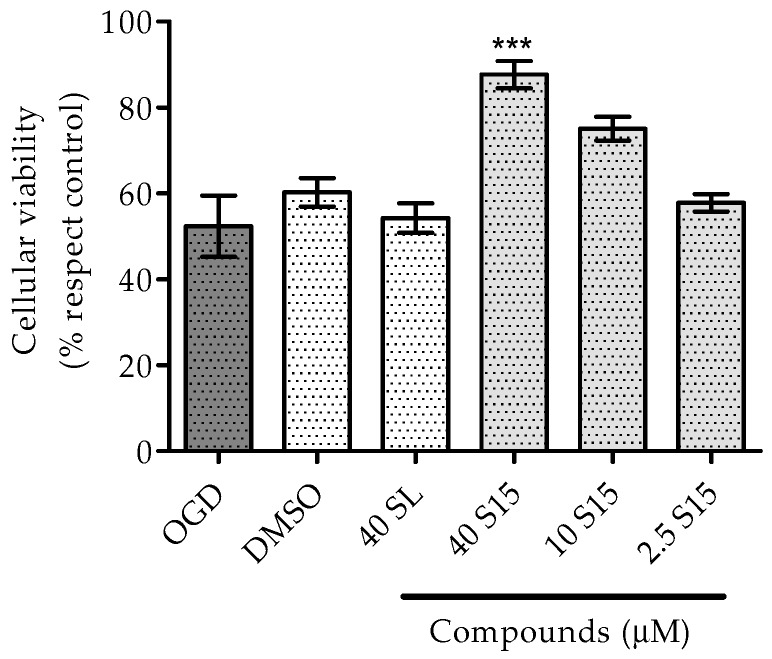
Neuroprotective activity of compounds against cell damage induced by chemical hypoxia and glucose deprivation. PC12 cells were cultured in oxygen and glucose deprivation conditions (OGD), specifically in the absence of glucose and in the presence of 600 µM of potassium cyanide (KCN) for 1 h, and then, normal culture medium was reperfused to the cells. After 4 h of the reperfusion start, some PC12 cells were treated with solvent control (DMSO), solasodine (SL) or different concentrations of the **S15** derivative. After 20 h of exposition to the reperfusion damage and treatment, cellular viability was determined by the MTT assay. Data are represented as the percent with respect to the untreated cells, representing the highest cell viability. Asterisks indicate statistical differences between the treatment and respective control condition (*** *p* < 0.001) according to one-way ANOVA and Tukey’s *post-hoc* test.

It has been reported that 17β-estradiol and other estrogenic steroids are associated with neuroprotective mechanisms in cerebral ischemia models [6,38,43]. Therefore, we studied the effects of some naturally-occurring steroids, as well as the synthetic spirosteroid analogs, in two in vitro models related to cerebral ischemia, by the MTT colorimetric assay [44]. The results are shown in Figure 1 and Figure 2.

As indicated in Figure 1, the treatment of PC12 cells with 20 mM of l-glutamate for 24 h caused a reduction of cellular viability with respect to untreated cells (Figure 1A). PC12 is a cell line derived from rat adrenal medulla pheochromocytoma, which synthesize glutamate and catecholamines and can be differentiated by nerve growth factor (NGF) to a sympathetic phenotype expressing neurites and excitability [45]. Glutamate exerts its toxic effects on differentiated and undifferentiated PC12 cells in a dose- and time-dependent manner, through two proposed main mechanism involving excitotoxicity and oxidative stress [46]. Although mRNA for NMDA receptor subunits are expressed by PC12 cells, the protein expression and functionally of this receptor are still under debate [46]. However, it is clear that PC12 cells express functional excitatory metabotropic glutamate receptor type I (mGluR1 and 5) [47], which could contribute to the glutamate-induced excitotoxicity. On the other hand, high extracellular concentrations of glutamate induce in PC12 cells an NMDA receptor-independent cytotoxicity caused by the inhibition of CySS uptake, which leads to GSH depletion and ultimately to the oxidative glutamate toxicity [46]. With respect to the natural steroids tested in our experiments, the treatment of glutamate-damaged PC12 cells with E3 (estriol), SL (solasodine) and T (testosterone) significantly induced the recovery of cellular viability (Figure 1A), while other endogenous steroids, E1 (estrone) and TDCA (taurodeoxycholic acid), did not show any effect in this model (Figure 1A). The observed anti-excitotoxic activity of E3 is in accordance with previous observations related to the neuroprotective properties of this estrogenic compound [48]. The potent antioxidant activity of estrogens has been related to the presence of a hydroxyl group in position 3 of the A ring and is also described as one of the main mechanism for its neuroprotective effects [22,49]. Taking into account the remarkable structural similarities and antioxidant capacity between estriol and estrone, we would expect a comparable anti-excitotoxic activity; however, our results show a lack of significant anti-excitotoxic activity of E1 (Figure 1A). The neuroprotective and anti-ischemic activity of T is less convincingly demonstrated, since some reports demonstrate neuroprotection induced by this sex hormone, while other studies revealed detrimental effects [50,51]. One possible explanation may involve the conversion of T to estrogen by aromatases and, therefore, the activation of neuroprotective estrogen-related signals [52]. The treatment of our glutamate-damaged PC12 cells with T significantly improves the viability of the cells (Figure 1A), which could be related to aromatase conversion in PC12 cells.

Despite the great amount of evidence showing the neuroprotective activity of endogenous steroids, the adverse effects of their uses in clinical trials has stimulated the research for new synthetic or natural therapeutic candidates [38,53]. In this sense, solasodine (SL) has shown neurogenic potential in PC12 cells [37], as well as in vivo anti-convulsive activity [36]. In our PC12 glutamate-induced excitotoxicity model, the treatment with SL significantly improved the cellular viability (Figure 1A). Furthermore, when glutamate-damaged PC12 cells were co-treated with the synthetic spirosteroid analog **S15**, the cellular viability was significantly increased with an EC_50_ of 10.97 µM (Figure 1A,B). In contrast, spirosteroid derivative **S9** did not significantly affect the glutamate-induced cell death (Figure 1A). These results suggest the importance and specificity of the substituent in position 3 in order to obtain the in vitro anti-glutamatergic activity (Figure 1 and Figure 2). Moreover, the carbonyl group at position 3 appears to be critical for this cellular activity, in combination with the hydroxyl and carbonyl groups at positions 5 and 6, respectively (Figure 1 and Figure 2).

Besides glutamate-induced excitotoxicity signaling, the mechanisms of oxidative imbalance and mitochondrial damage are also involved in the ischemic neuronal cell death [4,5]. Taking into account that SL and **S15** restored the cellular viability of glutamate-damaged PC12 cells, we further evaluated the in vitro activity of those steroids in PC12 cells exposed to cyanide-induced mitochondrial dysfunction. As indicated in Figure 2, among the two evaluated steroids, only the synthetic spirosteroid analog **S15** significantly protects PC12 cells from the mitochondrial damage induced by cyanide, in a dose-dependent manner (Figure 2). One of the neuroprotective mechanisms described in several nerve cells involves protein kinase B, PI3K and Akt kinases, which are known to be engaged in antiapoptotic and prosurvival activities. Specifically, it has been suggested that the PI3K/Akt pathway may regulate both NGF and BDNF-mediated neuronal survival [46,54]. However, in the scenario of PC12 cells exposed to oxygen and glucose deprivation, previous results have shown that overactivation of the antiapoptotic Akt kinase is detrimental rather than beneficial, highlighting the relevance of a balance in the activation status of Akt [55]. In this context, it has been demonstrated that adipose mesenchymal stem cells protect against glutamate-induced injury in PC12 cells by secreting neurotrophic factors, such as BDNF, through the involvement of the PI3-K/Akt and MAPK pathways [56]. In addition, smilagenin, a steroidal sapogenin from traditional Chinese medicinal herbs, stimulates BDNF mRNA transcription and attenuates Aβ(25–35)-induced neurodegeneration in primary cortical neurons and in a neuroblastoma cell line [57]. We could then hypothesize that the **S15** semi-synthetic sapogenin derivative exerts its protective effects on glutamate-damaged PC12 cells through the induction of neuroprotective signaling pathways, such as PI3-K/Akt, and probably the induction of neurotrophic factors. It is worth keeping in mind that, although PC12 is one of the most utilized cell lines for studies concerning the function of neurons, neuronal differentiation and neurotoxicity, primary cultures of cortical neurons are also widely used because they present all of the characteristics of living neurons activated by ionotropic glutamate receptor agonists [46]. Further studies in neuron primary cultures may provide more precise physiological information regarding the neuroprotective potential of Compound **S15** in excitotoxicity models. Altogether, this observed in vitro neuroprotective activity of Compound **S15** supports its role as a potential therapeutic candidate for neurological diseases involving mitochondrial dysfunction and excitotoxicity.

#### 2.2.2. Measurements of IL-1β and Nitric Oxide Levels

**Figure 3:**
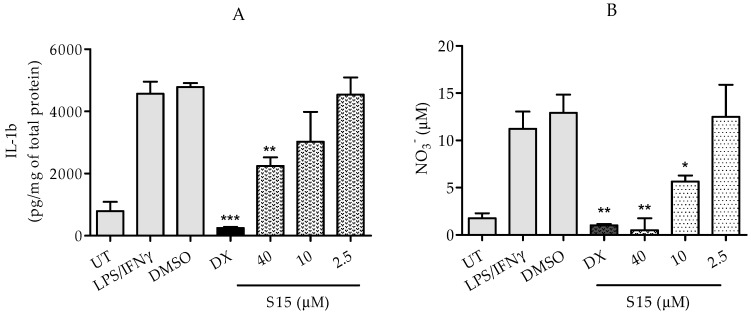
In vitro anti-inflammatory activity of the **S15** derivative in activated primary microglia. Microglia-enriched primary cultures were obtained from brains of adult male rats. Different concentrations of the **S15** derivative, solvent control (DMSO) or 10 µM of dexamethasone (DX) as the positive control of inhibition were applied to some microglia cultures, 30 min before the activation of microglia cells with lipopolysaccharide (LPS, 1 µg/mL) and murine recombinant interferon gamma (IFNγ, 20 U/mL). Untreated (UT) microglia cells were included as the negative control of inhibition. After 24 h of exposition or not to the stimulus and treatment, the IL-1β level was quantified by ELISA (**A**); and the content of nitrite was determined by the Griess assay (**B**). Asterisks indicate statistical differences between the treatment and respective control condition (* *p* < 0.05, ** *p* < 0.01 and *** *p* < 0.001) according to one-way ANOVA and Tukey’s *post-hoc* test.

The inflammatory response after brain tissue damage and ischemic neuronal cell death has been widely reported [4,5]. Therefore, we established a microglia primary culture, and we exposed the cells to LPS (1 µg/mL) and IFNγ (20 U/mL) in order to induce the release of pro-inflammatory molecules, as an experimental model of in vitro neuro-inflammation. The treatment with the synthetic spirosteroid **S15**, or dexamethasone as the inhibition control, was applied to the cells 30 min before the induction with LPS and IFNγ. After 24 h of treatment, nitrite and IL-1β concentrations were measured in the culture medium by the Griess assay and ELISA. The primary microglia were able to release significant levels of nitrite and IL-1β, in response to the stimulus with LPS/IFNγ and with respect to untreated cells (Figure 3A,B). As expected, the treatment with dexamethasone (10 µM) significantly decreased the high levels of nitrite and IL-1β, induced by LPS/IFNγ stimulus (Figure 3). The treatment with the analog **S15** inhibited the LPS/IFNγ-induced production of nitric oxide and IL-1β by microglia cultures, in a dose-dependent fashion (Figure 3). In vitro studies involving inflammatory stimulation with lipopolysaccharide, phorbol ester or interferon-gamma have demonstrated that 17β-estradiol dose-dependently decreases the phagocytic activity, inducible nitric oxide synthase increased expression and superoxide release of activated microglia, through a mechanism involving an estrogen receptor-dependent activation of MAP kinase [58,59]. Previous work has demonstrated that pre-treatment of ovariectomized rats with 17β-estradiol attenuates the activation of NF-κB and iNOS expression during post-ischemic reperfusion [60,61]. Therefore the modulation of NF-κB activity through hormone receptor-mediated signaling could be relevant for the mechanism of action of Compound **S15**, in the context of neuroprotection for ischemia/reperfusion, a complex disease where not only neuronal cell death, but also neuroinflammation takes place. On the basis of these in vitro results, the effects exerted by the synthetic spirosteroid **S15** on cell death and neuroinflammatory response suggest a potential in vivo neuroprotective effect.

#### 2.2.3. Rat Uterotrophic Assay

**Figure 4:**
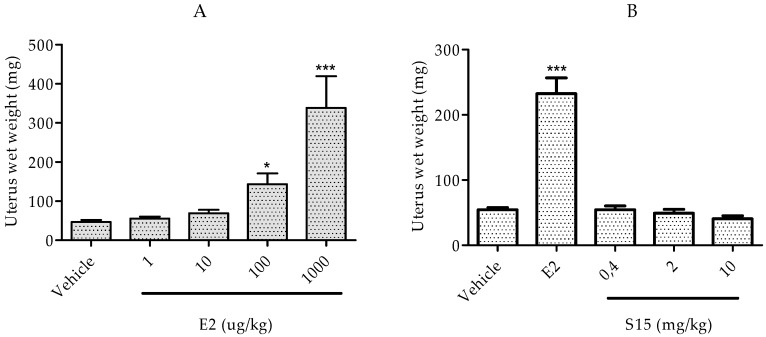
Uterine weight determined by the uterotrophic assay in immature female rats. Different doses of (**A**) E2 and (**B**) Compound **S15** or E2 (150 µg/kg) were administered to Post-natal Day (PND) 18 immature rats for three consecutive days. One day after the last dose, animals were sacrificed, and the uterus wet weight was measured. Asterisks indicate statistical differences between the treatment and respective control condition (* *p* < 0.05 and *** *p* < 0.001) according to one-way ANOVA and Tukey’s *post-hoc* test.

The estrogenic potential of a compound can be determined by the in vivo uterotrophic assay, which includes two possible variants. It is based on the principle that estrogens regulate the growth phase of the uterus in the physiological estrous cycle. We used one model that uses females in the sexually immature state before the activation of the hypothalamic-pituitary-gonadal axis and the synthesis of ovarian estrogens [24]. In order to evaluate the estrogenic potential according to OECD guidelines, immature female rats were administered with E2 (positive control) or Compound **S15**, and the uterus wet weight was determined. As we expected, there was an increase in the uterus wet weight of immature rats treated for three consecutive days with E2, in a dose-dependent manner (Figure 4A), showing that the model was successful in our conditions. In the case of Compound **S15** we did not observe any significant increase in the uterus wet weight in any of the three applied doses (Figure 4B). These results reveal a lack of estrogenic activity for Compound **S15** concerning the induction of uterine growth, which may represent a pharmacological advantage in terms of hormonal adverse side effects. However, the interaction of **S15** with classical nuclear or membrane-bound ER needs to be further studied by in vitro ER binding and transactivation assays, as has been suggested for several endocrine disrupting compounds [62]. The lack of a remarkable estrogenic effect of the **S15** analog, according to the uterotrophic assay in immature rats, may represent a pharmacological advantage in terms of hormonal adverse side effects.

## 3. Materials and Methods

### 3.1. Chemistry

All reagents were purchased from Merk Millipore and Panreac (Barcelona, Spain). The solvents, with technical quality American Chemical Society (ACS)-certified, were dried and distilled before use. The diosgenin was purchased from Sigma-Aldrich (Oakville, ON, Canada). Column chromatography was performed on Merk silica gel 60 (70–230 mesh ASTM, Merck, Barcelona, Spain). Analytical thin-layer chromatography (TLC) was carried out on silica pre-coated (0.25 mm) Merck silica gel GF254 (50 mm × 100 mm, Merck). The samples were developed with a vanillin solution of 1% in 50% perchloric acid. Melting points (mp) were recorded using an oven with the Electrothermal 9100 capillary apparatus, and the temperatures were uncorrected. The FTIR spectra were recorded in a Philips Analytical PU 9600 FTIR spectrophotometer in the 450–4500 cm^−1^ interval in KBr pellets at room temperature. NMR spectra were recorded on a Bruker DRX-400 spectrometer (Rheinstetten, Germany) operating at 400 MHz for ^1^H and 100 MHz for ^13^C. The samples were dissolved in deuterated chloroform, and the chemical shifts are expressed in ppm. The separation and purification process of the synthesized compounds by column chromatography (CC) was developed by using as the solvent a mixture of *n*-hexane/ethyl acetate (*v*/*v*) according to the derivatives.

#### 3.1.1. (25*R*)-5-Spirost-3β-ol-3-acetate (**6**)

Diosgenin 1 (10 g, 24 mmol) was dissolved in 20 mL of pyridine and 20 mL of Ac_2_O. After allowing the mixture to settle at room temperature for 24 h, it was poured into ice-cold water. The product was washed with diluted HCl and in the second step with abundant water to obtain 10.4 g of Compound **6** (95%). Physicochemical properties for this intermediary were reported in [39].

#### 3.1.2. (25*R*)-5,6-Epoxy-spirost-3β-ol-3-acetate (**7**)

After dissolving 10 g of Compound **6** (10.96 mmol) in 20 mL of chloroform, 3 g (17.53 mmol) of meta-chloro-perbenzoic acid were added at room temperature. The reaction mixture was stirred for 30 min, diluted with 25 mL of chloroform and washed with aqueous Na_2_SO_3_, aqueous Na_2_CO_3_ and water. The organic layer was dried and concentrated under vacuum to afford 8.64 g of solid Compound **7** (85%). Physicochemical properties for this intermediary were reported in [39].

#### 3.1.3. (25*R*)-Spirost-3β,5α-dihydroxy-6-one-3-acetate (**8**)

Seven grams (14.1 mmol) of Compound **7** were dissolved in 92.5 mL of acetone. Then, a solution of 7.5 g (74.7 mmol) of CrO_3_ in 16.2 mL of water was added with soft refluxing. After the reaction was finished, the solution was poured in water. The resulting solid was filtered, washed with water and re-crystallized from methanol. Five-point-eight grams of Compound **8** were obtained (80%). Physicochemical properties for this intermediary were reported in [39].

#### 3.1.4. (25*R*)-Spirost-3β,5α-dihydroxy-6-one (**S9**)

Five grams (10.2 mmol) of Compound **8** were dissolved in 390 mL of methanol. Subsequently, a solution of 4.6 g of NaHCO_3_ in 60 mL of water was added while refluxing for 3 h. After allowing the reaction mixture to settle at room temperature, it was poured in water, to obtain 4.35 g of Compound **S9** (95%), mp 272 °C (methanol). IR (KBr): ν_max_ 3396 (ν OH), 2953 (ν CH), 1703 (ν C=O), 1449 and 1380 (δ CH), 1055 (ν C=O), 977 (spiroketal); ^1^H-NMR (δ, ppm): 1.97/1.30 (m, 2H, H-1), 1.80/1.45 (m, 2H, H-2), 3.93 (m, 1H, H-3), 1.84/1.69 (m, 2H, H-4), 2.82/2.04 (m, 2H, H-7), 1.94 (m, 1H, H-8), 1.99 (m, 1H, H-9), 1.51/1.36 (m, 2H, H-11), 1.81/1.28 (m, 2H, H-12), 1.47 (m, 1H, H-14), 1.96/1.29 (m, 2H, H-15), 4.44 (m, 1H, H-16), 1.83 (m, 1H, H-17), 0.82 (s, 3H, CH_3_-18), 0.83 (s, 3H, H-19), 1.90 (m, 1H, H-20), 0.99 (d, 3H, CH_3_-21), 1.70/1.60 (m, 2H, H-23), 1.65/1.46 (m, 2H, H-24), 1.64 (m, 1H, H-25), 3.48/3.40 (m, 2H, H-26), 0.82 (d, CH_3_-27). ^13^C-NMR (δ, ppm: 30.4 (C-1), 29.8 (C-2), 67.2 (C-3), 36.5 (C-4), 80.7 (C-5), 211.7 (C-6), 41.9 (C-7), 36.7 (C-8), 44.6 (C-9), 42.4 (C-10), 21.3 (C-11), 39.5 (C-12), 41.1 (C-13), 56.1 (C-14), 31.6 (C-15), 80.5 (C-16), 62.0 (C-17), 16.4 (C-18), 14.2 (C-19), 41.6 (C-20), 14.5 (C-21), 109.3 (C-22), 31.3 (C-23), 28.8 (C-24), 30.3 (C-25), 66.9 (C-26), 17.1 (C-27).

#### 3.1.5. (25*R*)-Spirost-5α-hydroxy-3,6-dione (**S15**)

After dissolving 4 g (9 mmol) of Compound **S9** in 500 mL of acetone, 4 mL of Jones reagent (8 N-CrO_3_:5.33 g of CrO_3_ were dissolved in 4.4 mL of concentrate H_2_SO_4_, then water was added to obtain a 20-mL solution) were included drop-wise at room temperature. When the reaction was concluded, isopropanol was added until the mixture lost the orange color. After the solution was filtered by gravity, it was poured in water. The solid product was purified by column chromatography (1:1 hexane–EtOAc) affording 3.1 g of **S15** (81.5%), mp 283 °C (methanol). IR (KBr): ν_max_ 3345 (ν OH), 2953 (ν CH), 1713 (ν C=O), 1456 and 1376 (δ CH), 1057 (ν C-O), 980 and 899 (spiroketal); ^1^H-NMR (δ, ppm): 2.10/1.86 (m, 2H, H-1), 2.40 (m, 2H, H-2), 2.78/2.25 (m, 2H, H-4), 2.96/2.34 (m, 2H, H-7), 1.99 (m, 1H, H-8), 1.90 (m, 1H, H-9), 1.50 (m, 2H, H-11), 1.81/1.31 (m, 2H, H-12), 1.46 (m, 1H, H-14), 1.99/1.32 (m, 2H, H-15), 4.47 (m, 1H, H-16), 1.85 (m, 1H, H-17), 0.83 (s, 3H, CH_3_-18), 1.04 (s, 3H, CH_3_-19), 1.90 (m, 1H, H-20), 1.01 (d, 3H, CH_3_-21), 1.69 (m, 2H, H-23), 1.50 (m, 2H, H-24), 1.66 (m, 1H, H-25), 3.5/3.38 (m, 2H, H-26), 0.82 (d, 3H, CH_3_-27). ^13^C-NMR (δ, ppm): 31.8 (C-1), 37.3 (C-2), 210.2 (C-3), 41.9 (C-4), 82.7 (C-5), 210.4 (C-6), 44.8 (C-7), 36.8 (C-8), 44.7 (C-9), 43.1 (C-10), 21.3 (C-11), 39.5 (C-12), 41.0 (C-13), 56.0 (C-14), 31.6 (C-15), 80.4 (C-16), 62.0 (C-17), 16.4 (C-18), 13.9 (C-19), 41.6 (C-20), 14.5 (C-21), 109.3 (C-22), 31.3 (C-23), 28.8 (C-24), 30.3 (C-25), 66.9 (C-26), 17.1 (C-27).

### 3.2. Biological Activity Assays

#### 3.2.1. Treatments for PC12 Cell Cultures

The PC12 cell line was obtained from the American Type Culture Collection (Rockville, MD, USA) and maintained in a controlled environment at 37 °C and a 5% CO_2_ atmosphere. The cells were grown in RPMI-1640 medium supplemented with 5% fetal bovine serum (FBS), 10% horse serum and antibiotic/antimycotic solution (Sigma-Aldrich A5955). The culture medium was changed every 2 days, and the cells were seeded at a cellular density of 15,000 cells/mL in 96-well plates. To evaluate the effect of synthetic analogs of plant-derived spirosteroids, commercially available spirosteroids and endogenous steroids, on neurotoxic cell death related to cerebral ischemia, PC12 cells were exposed to an excitotoxic concentration of l-glutamate (20 mM) and co-treated with each natural and synthetic compound or vehicle (0.01% DMSO in culture medium) for 24 h. As another damaging condition closely related to ischemic neuronal death, PC12 cells were exposed to chemical hypoxia with potassium cyanide (KCN) (600 µM) in glucose-free culture medium for 1 h. Following 4 h of restoring the normal culture medium, without KCN, PC12 cells were treated with each natural and synthetic compound or vehicle (0.01% DMSO in culture medium) for 20 h. In our previous work, cytotoxicity experiments in PC12 cells were done to determine the limit of the non-cytotoxic dose for each compound [63]. A concentration below this value was chosen for the cyto-protective activity studies, and for comparative experiments, we worked with the same concentration for all compounds (40 µM or 4 µM of TDCA). In addition, the compound dose of 40 µM was selected for the glutamate-induced excitotoxicity experiments taking into account previous recommendations in drug discovery programs [64]. For the other in vitro experiments, the dose-response for each compound was designed from 40 µM and 4-fold decreases. All experiments were performed in phenol red-free medium and charcoal-stripped serum to remove steroids.

#### 3.2.2. MTT Assay

Cellular viability was evaluated by the colorimetric assay based on the measurement of the 3-(4,5-dimethylthiazol-2-yl)-2,5-diphenyltetrazolium bromide (MTT) reduction product [44]. MTT reagent can be converted to purple formazan crystals by mitochondrial dehydrogenases of viable cells. The high optical density at 570 nm detected after the complete reduction of MTT correlates with the fraction of viable cells [44]. After the desired incubation period with treatments and damaging conditions, the cell cultures were analyzed for MTT reduction. In brief, MTT was dissolved in PBS at 5 mg/mL and was 10-fold diluted in serum-free RPMI 1640 medium. After incubation of the cells with the compounds to be tested, the medium was aspirated, and MTT (0.5 mg/mL) containing serum-free medium was added. After an additional 3 h of incubation at 37 °C, isopropanol/HCl was added to each well, and the absorbance of solubilized MTT formazan products was measured at 570 nm.

#### 3.2.3. Microglia-Enriched Primary Cultures

Taking into account that neuro-inflammation is a common hallmark of many neurological diseases, such as cerebral ischemia, we established a microglia-enriched primary culture from adult rat brains, according to the procedure described by Moussaud and colleagues, with minor modifications [65]. Briefly, the brain of anesthetized and transcardially perfused, 2-month-old male Wistar rats was aseptically removed and carefully freed from the meninges. Only those brains properly exsanguinated were finely minced with a scalpel in the presence of the enzymatic solution (116 mM NaCl, 5.4 mM KCl, 26 mM NaHCO_3_, 1 mM NaH_2_PO_4_, 1.5 mM CaCl_2_, 1 mM MgSO_4_, 0.5 mM EDTA, 25 mM glucose, 1 mM cysteine and 20 U/mL of papain (all from Sigma-Aldrich)). After 90 min of incubation at 37 °C, 5% CO_2_ in continuous stirring, the enzymatic digestion was stopped with 20% heat-inactivated fetal bovine serum (FBS) in Hank’s balanced salt solution (HBSS) (Invitrogen, Karlsruhe, Germany), and the tissue suspension was centrifuged at 200× *g* for 7 min at room temperature. The resulting pellet was further digested with 0.5 mg/mL DNase I (Roche, Mannheim, Germany) in HBSS and incubated for 5 min at room temperature. After gentle disruption of the tissue with polished Pasteur pipettes of decreasing diameters, the homogenate was filtered through a 70-µm cell strainer (Becton Dickinson, Heidelberg, Germany). Then, the resulting cell suspension was centrifuged, and the cell pellet was resuspended in 20 mL of 20% stock isotonic Percoll (SIP) (GE Healthcare, Freiburg, Germany) (SIP = *v*/*v* ratio: 9/10 Percoll + 1/10 HBSS 10×) in HBSS. The cell suspension was carefully overlaid with 20 mL pure HBSS and centrifuged at 200× *g* for 20 min with slow acceleration and no brake. After this clarification step, the pellet with mixed glial cells was collected and washed once with HBSS. Finally, the cell pellet was resuspended in DMEM medium supplemented with glutamine 2 mM, 10 % FBS, 100 U/mL penicillin, 100 µg/mL streptomycin (all from Sigma-Aldrich) and 5 ng/mL of murine recombinant granulocyte and macrophage colony stimulating factor (GM-CSF) (315-03, Peprotech, Hamburg, Germany). The cell suspension from the cortical region of one brain was plated in one T75 flask (Greiner BioOne, Vilvoorde, Belgium), coated with poly-d-lysine (Sigma-Aldrich) and maintained in culture at 37 °C, 95% air/5% CO_2_ humidified incubator. The medium was changed twice a week until the cells became confluent (after approximately 2 weeks). Before the start of experiments, microglia were collected from the supernatant of confluent flasks and cultured for at least 3 days in medium without GM-CSF. The in vitro age of the cultures, in all of the experiments, was between 15 and 32 days in vitro (DIV).

For the NO and IL-1β induction experiments, the microglia cells were seeded at a density of 20,000 cells per well in 96-well plates, coated with poly-d-lysine (Sigma-Aldrich). Those cells were incubated for 30 min in DMEM medium supplemented with glutamine 2 mM, 10% FBS, 100 U/mL penicillin, 100 µg/mL streptomycin, with or without **S15** spirosteroid analogue at three different concentrations. Dexamethasone at 10 µM was used as a positive control for the inhibition of induced NO and IL-1β. After this pre-incubation with the compounds, microglia cells were stimulated with 1 µg/mL of lipopolysaccharide (LPS) from *Escherichia coli* strain 055:B5 (Sigma-Aldrich) and 20 U/mL of murine recombinant interferon gamma (IFNγ) for 24 h at 37 °C, 95% air/5% CO_2_.

#### 3.2.4. IL-1β and Nitric Oxide Assay

The amount of IL-1β released into the culture medium was measured using a sandwich ELISA system with antibodies specifics for rat IL-1β (Polyclonal Rat IL-1β/IL-1F2 Catalog Number AF-501-NA and Polyclonal Rat IL-1β/IL-1F2 Biotinylated Catalog Number BAF501, R&D Systems, Minneapolis, MN, USA), following the manufacturer’s instructions. Culture supernatants were collected 24 h after LPS/IFNγ treatment and stored at −80 °C until assayed for IL-1β content.

NO production was assessed by the Griess reaction. Briefly, culture supernatants were collected 24 h after LPS/ IFNγ stimulation and incubated with equal volumes of Griess reagent for 10 min at room temperature, in the dark. Optical density was measured at 540 nm using a microplate reader (Polarstar Omega, BMG Labtech, Offenburg, Germany). Nitrite concentration was calculated from a sodium nitrite standard curve.

#### 3.2.5 Immature Rat Uterotrophic Assay

Post-natal day (PND) 11 female Wistar rats were purchased from CENPALAB (Havana, Cuba) and allowed to acclimate for 7 days. Each group of six pups was housed in a polycarbonate cage and fed ad libitum with standard CMO 1000 diet CENPALAB (Havana, Cuba). The temperature of the environment was set at 20–24 °C with 40%–60% relative humidity and a 12-h light-dark cycle. E2 (1–1000 μg/kg) and Compound **S15** (0.4 or 10 mg/kg) were administered subcutaneously for 3 days to immature rats at PND 18. All experimental protocols were approved by the ethics committee for animal research, Havana University, Havana, Cuba.

### 3.3. Statistical Analysis

All experimental groups were analyzed with the D’Agostino and Pearson omnibus normality test to determine the Gaussian distribution of the data. Significant differences between groups were determined by 1-way ANOVA, followed by Tukey’s post hoc analysis with the GraphPad Instat program, Version 5, for Windows© (San Diego, CA, USA, www.graphpad.com). All data, at the 95% confidence interval, are expressed as the means ± standard error of the mean (SEM) from 3 independent experiments. Asterisks indicate statistical differences between the treatment and respective control condition (* *p* < 0.05, ** *p* < 0.01 and *** *p* < 0.001).

## 4. Conclusions

Several naturally-occurring steroids were biologically evaluated in a cellular model of excitotoxicity. Among them, E3, T and SL showed a neuroprotective activity against excitotoxicity, but not against the glucose deprivation and cyanide-induced cell death. In parallel, four semi-synthetic spirosteroid analogues were synthesized and evaluated as therapeutic candidates in the same two cellular models of damage (glutamate-induced excitotoxicity and glucose deprivation and cyanide-induced cell death) and also in neuro-inflammation induced in primary microglia. The specific chemical features of the synthetic spirosteroid **S15** may provide information that could be useful for the design of novel neuroprotective agents, using a sapogenin scaffold. Moreover, the **S15** derivative does not have a strong estrogenic effect, according to the uterotrophic assay in immature rats, which may give further advantages in the pharmacological use of this family of compounds. Further studies to improve and fully characterize the in vivo neuroprotective activity of this class of compounds need to be investigated.

## Supplementary Materials

Supplementary materials can be accessed at: http://www.mdpi.com/1420-3049/21/8/992/s1.
