# Supplementary material for: In Vitro Neuroprotective and Anti-Inflammatory Activities of Natural and Semi-Synthetic Spirosteroid Analogues
Source: Molecules. 2016 Jul 29;21(8):992. doi: 10.3390/molecules21080992 (PMC6274191; doi:10.3390/molecules21080992)
Supplement: Supplementary file 1 [file molecules-21-00992-s001.pdf]

Supplementary Material: In Vitro Neuroprotective and Anti-Inflammatory Activities of Natural and Semi-Synthetic Spirosteroid Analogues

Laura García-Pupo, Armando Zaldo-Castro, Vassiliki Exarchou, Juan Enrique Tacoronte-Morales, Luc Pieters, Wim Vanden Berghe, Yanier Nuñez-Figueredo and René Delgado-Hernández

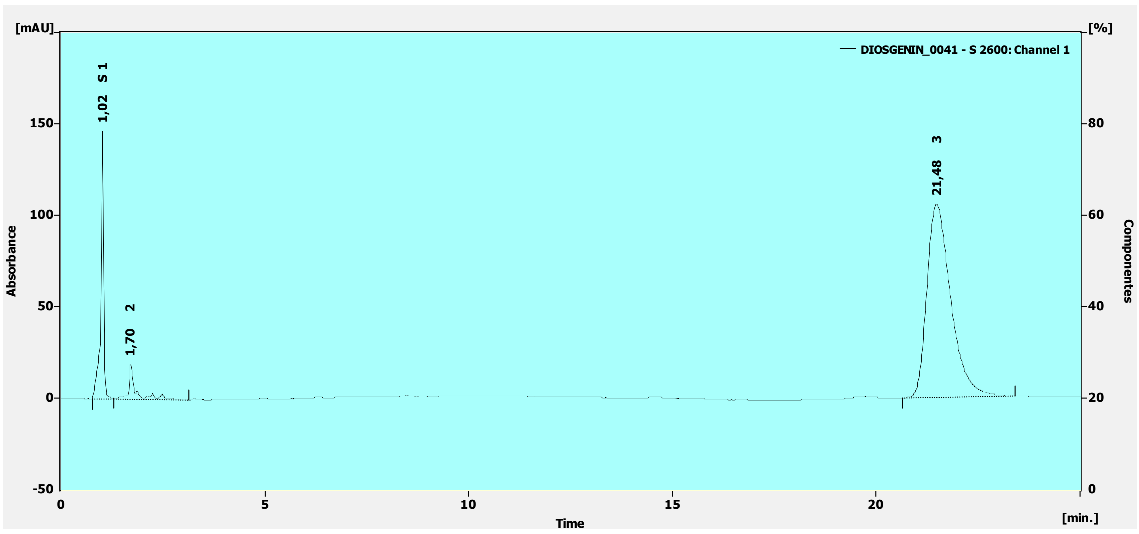

(**A**)

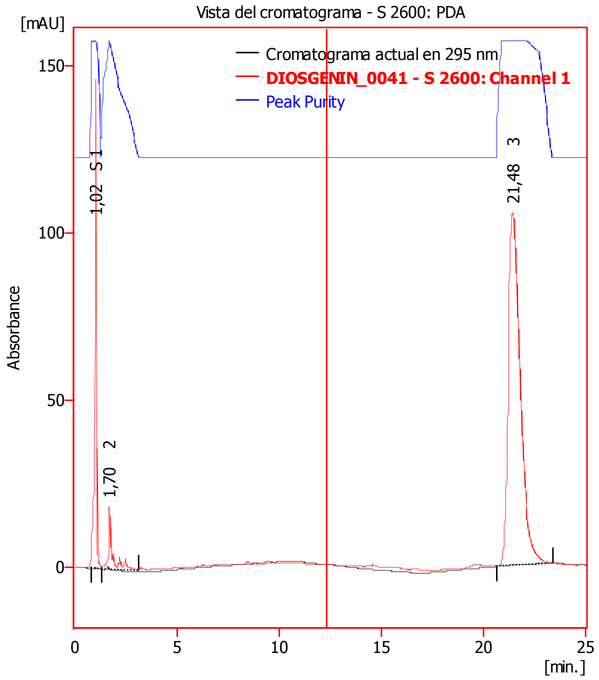

(**B**)

Figure S1. Relative quantification of Diosgenin purity by HPLC. (**A**) Chromatogram of the HPLC run and (**B**) HPLC chromatogram with a photodiode array(PDA) analysis of peak purity.

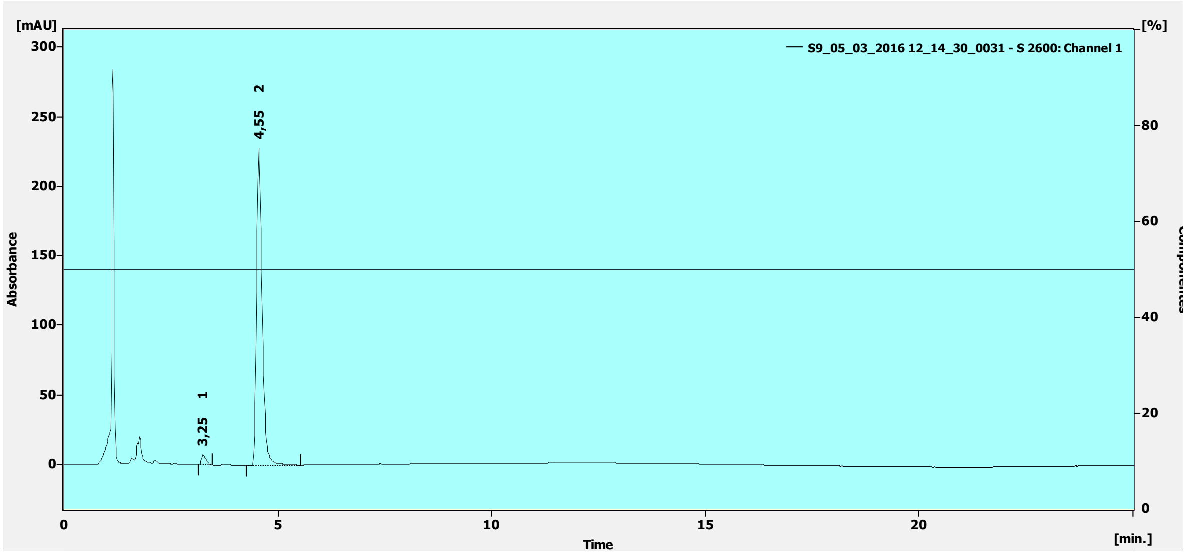

(**A**)

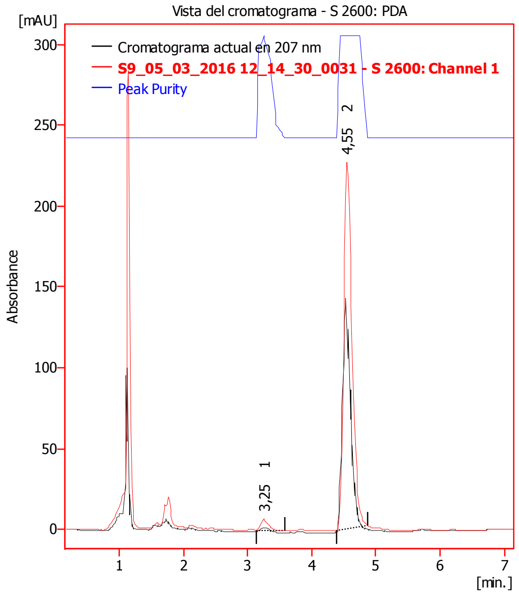

(**B**)

Figure S2. Relative quantification of compound **S9** purity by HPLC. (**A**) Chromatogram of the HPLC run and (**B**) HPLC chromatogram with a photodiode array(PDA) analysis of peak purity.

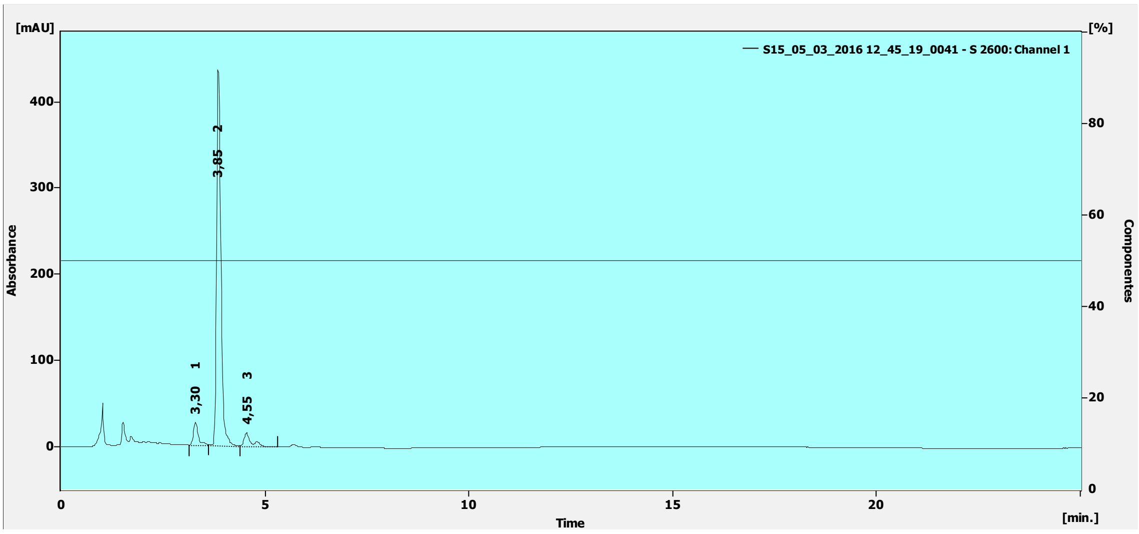

(**A**)

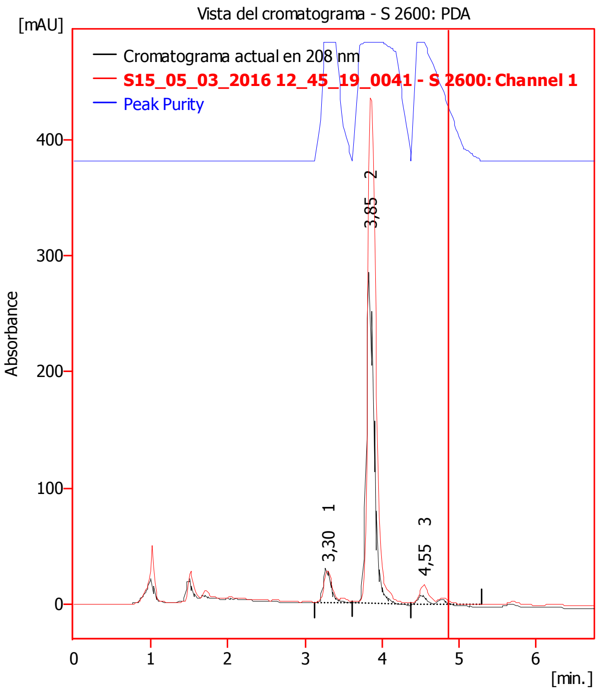

(**B**)

Figure S3. Relative quantification of compound **S15** purity by HPLC. (**A**) Chromatogram of the HPLC run and (**B**) HPLC chromatogram with a photodiode array(PDA) analysis of peak purity.

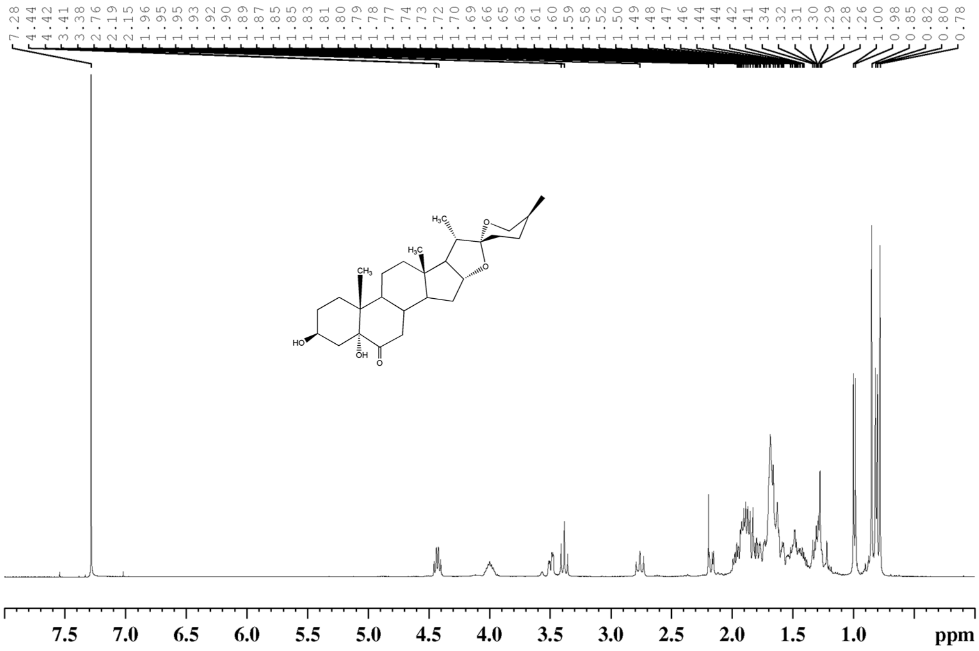

(**A**)

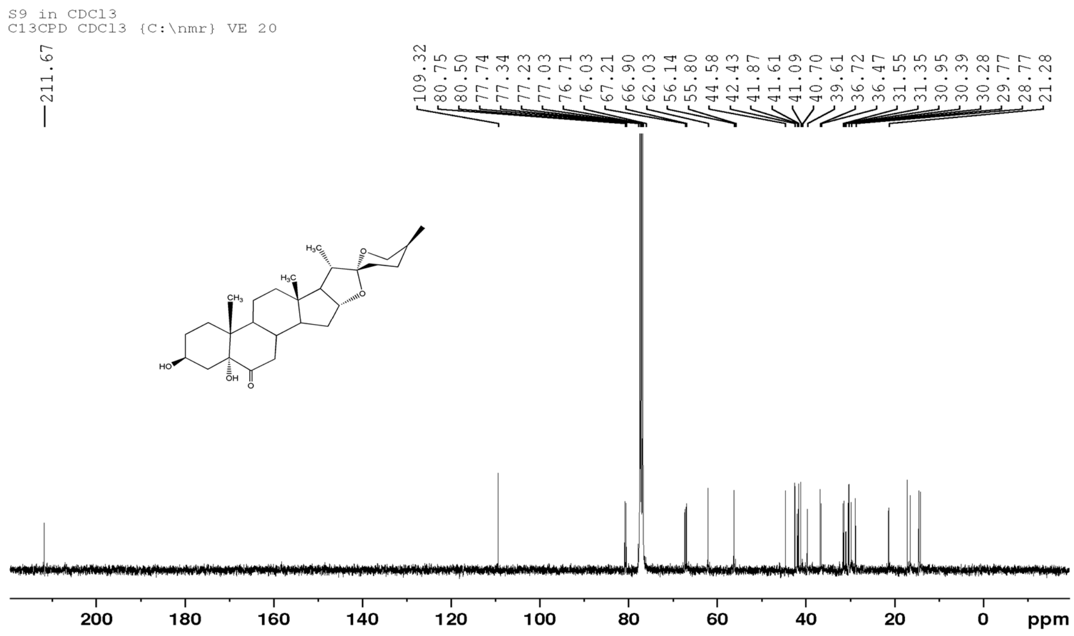

(**B**)

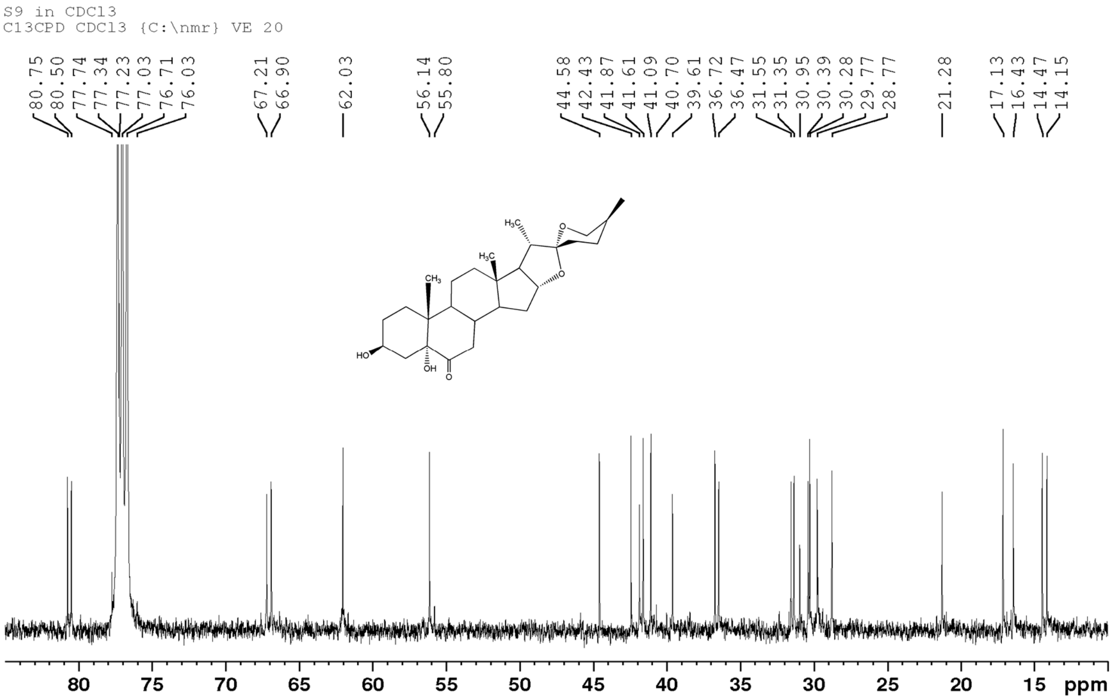

(**C**)

Figure S4. NMR spectroscopic data for compound **S9**. (**A**) ^{1}H-NMR; (**B**) and (**C**) ^{13}C-NMR.

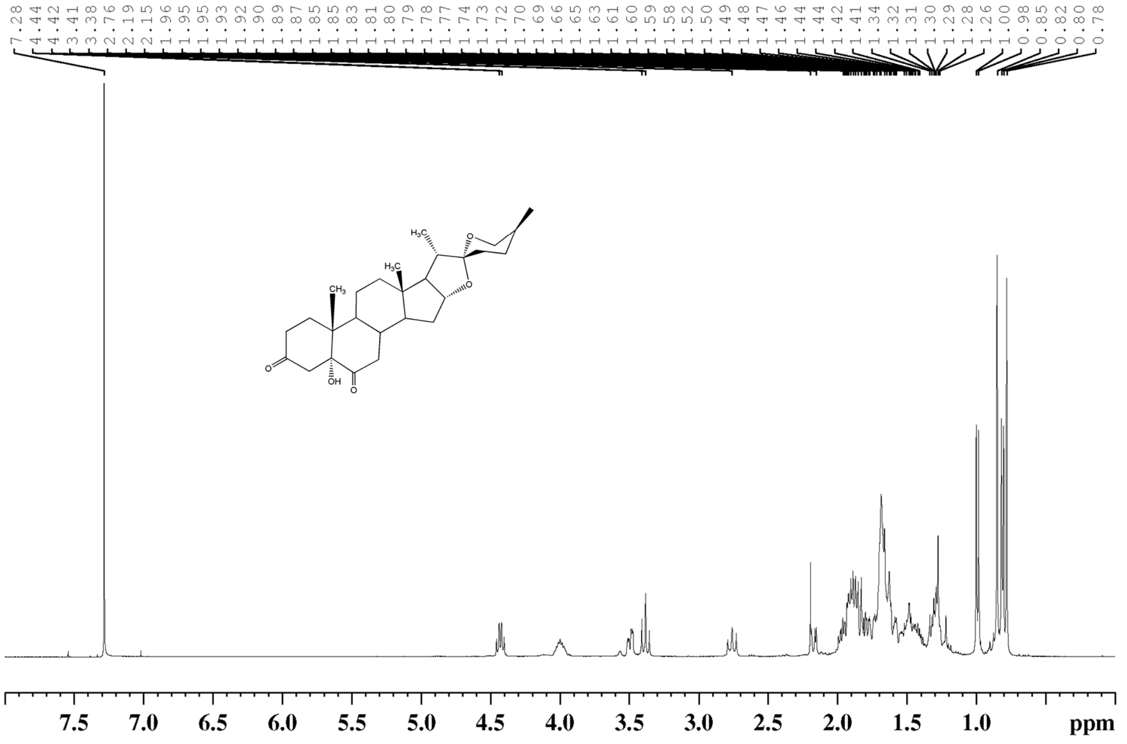
7.28 4.44 4.42 3.41 3.38 2.76 2.19 2.15 1.96 1.95 1.95 1.93 1.92 1.90 1.89 1.87 1.85 1.85 1.83 1.81 1.80 1.79 1.78 1.77 1.74 1.73 1.72 1.70 1.69 1.66 1.65 1.63 1.61 1.60 1.59 1.58 1.52 1.50 1.49 1.48 1.47 1.46 1.44 1.44 1.42 1.41 1.34 1.32 1.31 1.30 1.29 1.28 1.26 1.00 0.98 0.85 0.82 0.80 0.78
H3C
H3C
CH3
O
O
OH
7.5 7.0 6.5 6.0 5.5 5.0 4.5 4.0 3.5 3.0 2.5 2.0 1.5 1.0 ppm

(**A**)

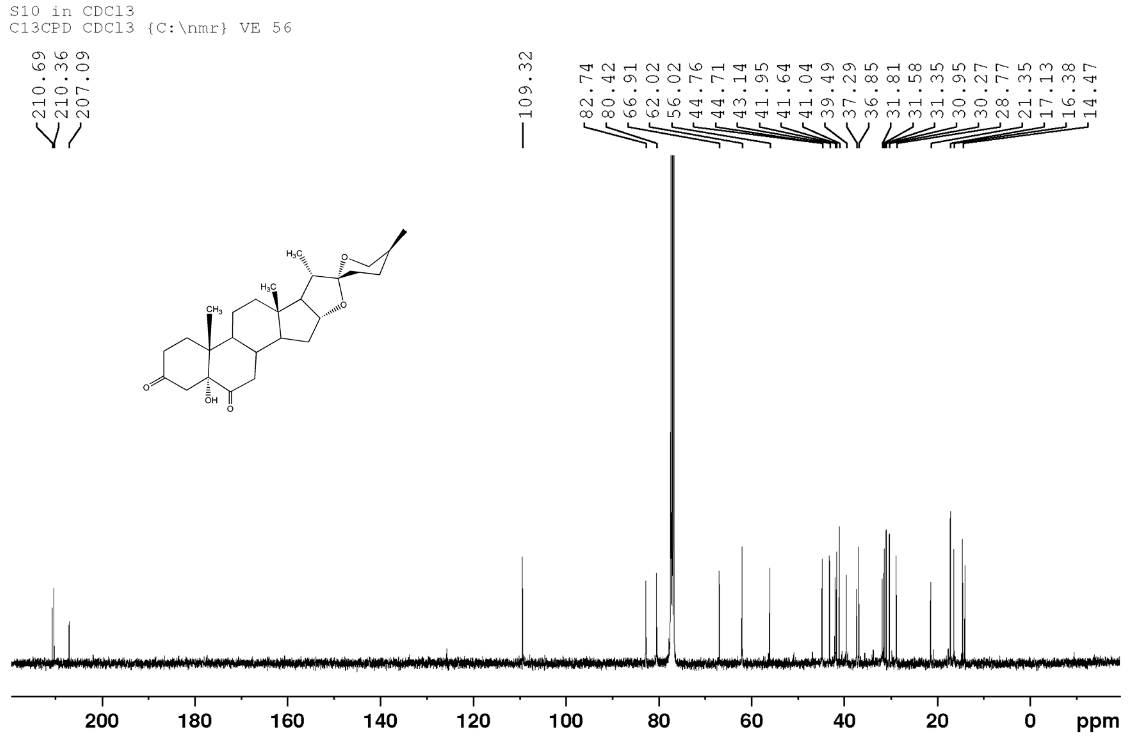
S10 in CDCl3
C13CPD CDCl3 {C:\nmr} VE 56
210.69 210.36 207.09
109.32
82.74 80.42 66.91 62.02 56.02 44.76 44.71 43.14 41.95 41.64 41.04 39.49 37.29 36.85 31.81 31.58 31.35 30.95 30.27 28.77 21.35 17.13 16.38 14.47
H3C
H3C
CH3
O
O
OH
200 180 160 140 120 100 80 60 40 20 0 ppm

(**B**)

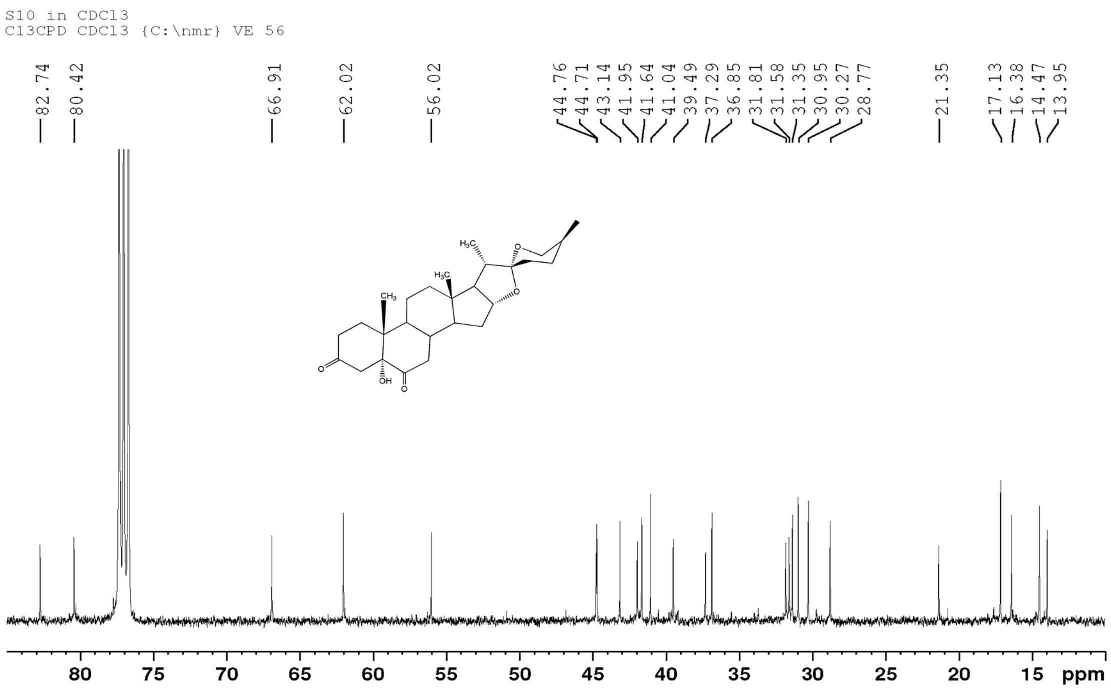

(**C**)

Figure S5. NMR spectroscopic data for compound **S15**. (**A**) ^{1}H-NMR; (**B**) and (**C**) ^{13}C-NMR.
